# Triple P-Positive Parenting Program for Mothers of ADHD Children

**Published:** 2014

**Authors:** Asma Aghebati, Banafsheh Gharraee, Mitra Hakim Shoshtari, Mahmood Reza Gohari

**Affiliations:** 1 PhD Student, Tehran Institute of Psychiatry, School of Behavioral Sciences and Mental Health, Iran University of Medical Sciences, Tehran, Iran.; 2 Associate Professor, Department of Clinical Psychology, Tehran Institute of Psychiatry, School of Behavioral Sciences and Mental Health, Iran University of Medical Sciences, Tehran, Iran.; 3 Child and Adolescent Psychiatrist, Mental Health Research Center AND Tehran Psychiatric Institute, School of Behavioral Sciences and Mental Health, Iran University of Medical Sciences, Tehran, Iran.; 4 Associate Professor, Department of Statistics & Mathematics, Hospital Management Research Center, Iran University of Medical Sciences, Tehran, Iran

**Keywords:** Attention Deficit Hyperactivity Disorder (ADHD), Anxiety, Behavioral Problems, Depression, Parenting Style

## Abstract

**Objective:** Attention deficit hyperactivity disorder (ADHD) is a chronic, highly prevalent neurodevelopmental disorder which affects 9% of school-age children. Triple P-Positive Parenting Program is an evidence-based parenting program reported to be useful in the management of this disorder. The aim of this randomized controlled trial was to evaluate the effectiveness of Triple P in mothers of ADHD children.

**Methods:** In this study, 30 mothers with ADHD children aged between 6 to 10 were randomly assigned to two groups (15 participants in each group). Parenting style, mother-child relationship, maternal depression, anxiety and stress, and children’s behavioral problems were evaluated. The intervention group received 120 minute sessions for 5 weeks and 15-30 minute telephone contacts for 3 weeks while no intervention was done for the control group.

**Results: **Analysis of covariance revealed that mothers of the Triple P group showed significant (p < 0.01) improvements in parenting style, mother-child relationship, and considerable decrease in depression, anxiety and stress. Women trained in the Triple P group also reported significantly lower rates of child misbehavior than women of the control group.

**Conclusion:** Triple P-Positive Parenting intervention is effective and acceptable for mothers of ADHD children.

**Declaration of interest:** None.

**Clinical Trial Registration-URL: **http://www.irct.ir. Unique identifier: IRCT201111288234N1.

## Introduction

Attention deficit hyperactivity disorder (ADHD) is a common disorder in school-age children (1). It causes the individuals many functional disabilities throughout their life; it typically emerges during the preschool years, and persists through adolescence and into adulthood for many afflicted individuals (2).

Children with ADHD are inattentive, impulsive, and overactive and all of these characteristics challenge parents in effectively managing children’s behaviors, and they may develop a poor monitoring and inconsistent or punitive discipline strategy. This type of parenting predicts a lot of negative adolescent and adult outcomes, including alcohol and substance abuse, delinquency, and academic failure (3).

For treatment of ADHD, pharmacological interventions (most prominently stimulant medications), behavioral interventions, parent training, and contingency management in the classroom are considered the best-supported. These interventions result in significant benefits for children with ADHD in many domains of functioning (4, 5). Studies of parent training for ADHD have demonstrated improvements in ADHD symptoms, and co-occurring oppositional problems and impairment in children (6-9). Parent training also improves parental functioning (e.g., decreased stress, enhanced competence) (10, 11).

Triple P is a parent training program that is designed to prevent severe behavioral, emotional, and developmental problems in children by enhancing the knowledge, skills, and confidence of parents. Triple P incorporates five levels of intervention of increasing strengths that help parents of children from birth to age 12. Level 1 provides all parents with access to information about parenting using print and electronic media with the aim of increasing community awareness of parenting resources, and to encourage parents to participate in programs. Level 2 consists of 1 or 2 sessions for providing anticipatory developmental guidance to parents of children with mild behavior difficulties, with the aid of tip sheets and videotapes about specific parenting strategies. Level 3 targets children with mild to moderate behavioral problems and includes 4 sessions about active skills training. Level 4 is a program for parents of children with more severe behavioral difficulties. Level 5 interventions include enhanced behavioral family interventions in more severe cases (e.g., relationship conflicts, parental depression, or high levels of stress). This strategy recognizes that parents have different needs and desires regarding the type, intensity, and the mode of assistance they require (12).

Although a few researchers examined the effect of Triple P on ADHD children in Iran, none of them assessed mother-child relationship and they did not control the effect of the medications on children. The aim of this study is to assess the effectiveness of Triple P on the parenting style, mother-child relationship, maternal depression, anxiety and stress, and behavioral problems of ADHD children.

## Materials and Methods

The effectiveness of Triple P was tested by means of a randomized controlled trial (examining 2 groups). The study population consisted of mothers introduced by several child psychiatrists in psychiatric clinics in Tehran, Iran, in 2010 and 2011. Participants who met all of the criteria for this research were randomly placed into 2 groups (15 people in each group). All of the participants were female and their mean age was 33.21 ± 5.25 years in the intervention group and 34.46 ± 9.30 years in the control group. 

Each family met the following inclusion criteria: child’s age between 6 and 10 years (early school age); interested and cooperative family; diagnosis of ADHD by both a child and adolescent psychiatrist, and an interview based on Diagnostic and Statistical Manual of Mental Disorders, 4^th^ Edition (DSM-IV) by a clinical psychologist. All children or mothers taking new medication in the last month or in regular contact with another professional for psychological problems were excluded. All of the children (in both groups) used methylphenidate with the 10-20 mg dosages, at least for one month before the research and there was no change in the type and dosage of the drug during the intervention. 

The interventions were described to help parents better manage everyday family life. Written informed consents were obtained from all participating families. Participants were asked to complete questionnaires at two times; at pre-test (first time, prior to the intervention), and post-test (second time, after the completion of the training). The control group completed the questionnaires at the same time as the Triple P group. After post-test, the control group also participated in this intervention. This study received ethical committee confirmation from Iran University of Medical Sciences. The Clinical Trial Registration number of the present study is 201111288234N1.

***Intervention conditions***

The Level 4 of Triple P was used in this research. This is an 8-session group parenting program for parents of children with severe behavioral difficulties (5 workshop sessions for 2 hours and 3 telephone sessions for 15-30 minutes for each participant) (12).

***Instruments***

*Parenting Scale* (PS)

This scale is a 30-item questionnaire measuring 3 dysfunctional discipline styles: laxness (permissive discipline), over-reactivity (authoritarian discipline), and verbosity (overly long reprimands or reliance on talking). Each item has a 7-point scale which makes the total score. This questionnaire has good internal consistency and test–retest reliability (13). It has been used in Iran and has high reliability (0.69 for laxness, 0.77 for over-reactivity, and 0.72 for verbosity) and validity (14).

*The Depression Anxiety Stress Scale-42* (DASS)

This scale is a 42-item questionnaire assessing symptoms of depression, anxiety, and stress in adults. There are 12 items for each one which range from 0 to 3. DASS has 3 scores for depression, anxiety, and stress. This questionnaire has high reliability and internal consistency reliability (15). Iranian researchers reported acceptable reliability (0.77 for depression, 0.66 for anxiety, and 0.76 for stress) and validity for this scale (16).

*Parental bounding instrument* (PBI)

This instrument is a 25-item questionnaire, including 12 ‘care’ items and 13 ‘overprotection’ items. This questionnaire reported 2 scores. Both of them had good split-half reliability (0.78 for care and 0.74 for overprotection) and concurrent validity in different studies including in Iran (17, 18).

*Child Behavior Checklist (CBCL)*

CBCL is a widely used 118-item inventory from which scores can be computed for the full scale, externalizing behaviors, and internalizing behaviors. The full score was used in this study. The scale has good test–retest reliability and construct validity (19). This checklist was standardized in Iran and used in different studies (20).

*Client Satisfaction Questionnaire (CSQ)*

This measure is an adaptation of the Therapy Attitude Inventory which examines consumer satisfaction with this program (21). Its internal consistency and validity are high (22, 23). This questionnaire was used for the first time in Iran and its Persian version also had good reliability (Cronbach's alpha = 0.85).

***Statistical analysis***

Data are expressed as means (± SD). A series of multivariate analysis of covariance (MANCOVA) and analysis of covariance (ANCOVA) were used to compare the groups before and after the intervention. The pre-test scores were used as covariate variables. Values of p < 0.001 were considered statistically significant. All of these analyses were performed using SPSS for Windows (version 19; SPSS Inc., Chicago, IL, USA).

## Results

In the present study, 30 families were enrolled, but 27 (14 intervention and 13 controls) participants completed the study. The mean child age was (mean ± SD) 7.7 ± 1.5 years in the intervention group and 8.3 ± 1.2 years in the control group. There were 16 boys and 11 girls. ANOVA showed that there were no significant differences between groups regarding variables of demographic and family background (Table 1).

***Attrition***

Of the 30 families who were assigned to the two groups, 27 (90%) completed the post-test. There were no significant differences in any outcome measures between participants who completed post-tests versus those who did not. Of the 3 families who did not complete the post-test, 1 was an intervention group family (6.6%) and 2 (13.3%) were control group families. Chi-square analysis confirmed there was no significant difference in attrition rates across groups.

***Intervention effects***

Table 2 shows the means (± SD) of mother-reported measures at pre- and post-intervention. In relation to all measures, MANCOVA scores were significant. Then, a series of ANCOVA were calculated.

After the intervention the mean of parenting style (2.29), maternal depression (17.35), anxiety (21.28), stress (17.57), overprotection of child (13.28), and child’s misbehavior (36.35) decreased significantly, and the mean of care of child (21.92) increased. These results mean that Triple P can change all of the variables (parenting style, mother-child relationship, maternal depression, anxiety, stress, and child’s misbehavior) significantly. 

***Client satisfaction***

The maximum and minimum scores of the questionnaire were 89 and 51, respectively. The mean client satisfaction score was 76.57 (± 11.98). This finding indicates that parents were satisfied with the program on the whole.

## Discussion

The level 4 Triple P is a parent-mediated intervention that aims to improve positive parent–child interactions and parenting behaviors in order to reduce behavioral problems of children with ADHD. It has been shown to be effective in improving parenting style and child mental health (22, 24, 25). This program includes 8 sessions of intervention for parents of children with more severe behavioral difficulties (12).

The main findings of this study was that mothers of the Triple P group showed significant improvements in parenting style, mother-child relationship, and decrease in maternal depression, anxiety, stress, and child’s misbehavior.

Similar to this study, researchers show that the group Triple P is a cost-effective method of intervention that offered parents a way of increasing support and reducing social isolation, and provides additional learning experiences through sharing ideas and information, and through modeling positive behaviors (26). Sanders et al. showed that group Triple P is effective in promotion of parenting style and parental satisfaction, decreasing parental distress and parental conflict, and improvement of child misbehaviors in the families with ADHD children. Furthermore, parents reported high levels of consumer satisfaction similar to our study (27). In different studies, group Triple P has resulted in significant reductions of dysfunctional parenting practices, marital conflict, parental stress and depression, as well as significant improvements in marital satisfaction in families with ADHD children (28, 29). In a meta-analysis, the level 4 of Triple P had moderate to large effects on behavioral problems of ADHD children, which lasted for 6 to 12 months )^30^). Petra and Kohl suggested that this program is acceptable to parents (31). Their study demonstrates the effectiveness of this program in increasing parenting style, reducing child behavior problems, and preventing maltreatment recidivism. Low attrition rate indicates that parents found the program acceptable and a good fit for their needs. Participants believed that these techniques had helped them to become better parents and this demonstrates the acceptability of this intervention (32, 33). Although there are few researches about the effect of Triple P on mothers of ADHD children in Iran, the results of these studies are consistent with this article and improving parenting style, decreasing the maternal depression, anxiety, stress, and children’s misbehavior (34-36).

**Table 1:** Socio-demographic characteristics of the two groups

**Variables**	**Triple P (n = 14)**		**Control group (n = 13)**		**p**
	**Mean**	**(± SD)**	**Mean**	**(± SD)**	
**Age of child (year)**	7.7	(± 1.5)	8.30	(± 1.2)	0.708
**Age of mother (year)**	33.21	(± 5.25)	34.46	(± 9.30)	0.713
	**N**	**%**	**N**	**%**	
**Gender of child**					0.445
**Male **	9	64.3	7	53.8	
**Female**	5	35.7	6	46.2	
**Education of mother**					0.256
**Elementary school**	3	21.4	4	30.8	
**High school**	7	50.0	4	30.8	
**University**	4	28.6	5	38.5	
**Family income (Rials)**					0.686
**0-5000000**	7	50.0	3	23.1	
**5000000-10000000**	5	35.7	6	46.2	
**More than 10000000**	2	14.3	4	30.8	

**Table 2:** Analysis of covariance between Parenting Scale, depression, anxiety, stress, overprotection, care, and Child Behavior Checklist scores in Triple P and control groups

**Variables**	**Triple P** ^‡^ ** (n = 14)**		**control group (n = 13)**		**ANCOVA** ^†^	**p**
	**Pre-test**	**Post-test**	**Pre-test**	**Post-test**		
	**Mean (± SD)**	**Mean (± SD)**	**Mean (± SD)**	**Mean (± SD)**		
**Parenting Scale**	03.40 (± 0.80)	02.29 (± 0.70)	03.40 (± 1.22)	03.24 (± 1.17)	7.48	0.007
**Depression**	31.14 (± 5.93)	17.35 (± 4.82)	32.23 (± 4.31)	31.23 (± 4.58)	3.72	0.000
**Anxiety**	33.78 (± 5.57)	21.28 (± 6.80)	32.84 (± 5.08)	31.92 (± 4.62)	5.50	0.002
**Stress**	33.57 (± 5.25)	17.57 (± 5.66)	32.30 (± 4.75)	30.61 (± 4.95)	5.27	0.000
**Overprotection**	22.57 (± 6.03)	13.28 (± 2.16)	21.15 (± 6.09)	21.61 (± 6.41)	0.45	0.001
**Care**	16.35 (± 4.03)	21.92 (± 5.39)	16.69 (± 4.06)	16.38 (± 4.40)	1.63	0.002
**Child Behavior Checklist**	52.64 (± 1.40)	36.35 (± 1.14)	55.38 (± 1.30)	56.61 (± 1.47)	8.76	0.000

† Analysis of covariance;

‡ Positive parenting program

Overall, child behavioral problems have a negative impact on the ability of parents to undertake care-giving tasks, and these behavioral problems compound the difficulties experienced by parents. It is assumed that by reducing child behavioral problems, the burden of care is reduced for parents and they are more readily able to undertake their roles (23). Triple P enhances the skills, knowledge, self-sufficiency, confidence, and resourcefulness of parents. Furthermore, this program provides safe, nurturing, nonviolent, engaging, and low-conflict environments for children. These effects promote parental abilities and children's behavioral competencies through positive parenting practices (37). Therefore, Triple P can help parents in the better management of their children’s behavioral problems, especially for mothers of ADHD children. 

The limitation of this research was that results were based on parents' reflections and perceptions regarding parenting and child behavior problems, and no objective measurements or indices were used to investigate it. It is also possible that results are biased by the fact that parents were the only source of information. 

## Conclusion

The findings of this study revealed that Triple P is an effective program in improving parenting style, mother-child relationship, and decreasing maternal depression, anxiety, stress, and children’s misbehavior. These results can be used in treatment plans for ADHD children and their families, especially in Iran. Findings of this study provide support to previous empirical findings on the effectiveness of Triple P and also provide important knowledge on the cultural transferability of this program in Iran (11).

## Authors' contributions

AA, BG, and MHS conceived and designed the evaluation and helped to draft the manuscript. AA and BG collected the clinical data. AA and MRG interpreted the clinical data, performed the statistical analysis, and drafted the manuscript. All authors read and approved the final manuscript.
